# Precisely Integrated Mesoporous Anode Enabling Fast Pseudocapacitive Sodium-Ion Storage

**DOI:** 10.1021/acscentsci.5c00616

**Published:** 2025-08-18

**Authors:** Shuang Li, Jiecheng Chen, Xin Miao, Xu Wen, You Zhou, Bingxian Chu, Wendi Wang, Yanyan Yu, Ziyang Guo, Kun Lan

**Affiliations:** † College of Energy Materials and Chemistry, College of Chemistry and Chemical Engineering, 12576 Inner Mongolia University, Hohhot 010021, P. R. China; ‡ Department of Materials Science and Engineering, 255310 Southern University of Science and Technology, Shenzhen 518055, P. R. China

## Abstract

Sodium-ion batteries (SIBs) are considered potential alternatives to lithium-ion batteries (LIBs) due to the abundant resources and low sodium cost. The rational nanostructural design for anode materials plays a crucial role in SIBs. TiO_2_, as a common electrode material, suffers from the drawbacks of low specific surface area and poor conductivity. To overcome these limitations, we propose a strategy combining solvent evaporation-induced self-assembly and chemical oxidative polymerization to construct an ultrathin polypyrrole (PPy)-coated mesoporous TiO_2_ microsphere (meso-TiO_2_@PPy) core–shell structure. The combination of the mesoporous structure and the conductive coating endows the micrometer-sized TiO_2_ spheres with high specific surface area, excellent conductivity, and abundant sodium-ion diffusion pathways, leading to a dominant pseudocapacitance (94%) of total charge storage. Remarkably, such integration allows for a high reversible capacity of 160.6 mAh g^–1^ at 1 A g^–1^, good rate performance, and stable cycling performance (capacity retention of 80.8% after 2000 cycles). Our research provides a pathway for the design of compositive anode materials for high-performance SIBs.

## Graphical abstract

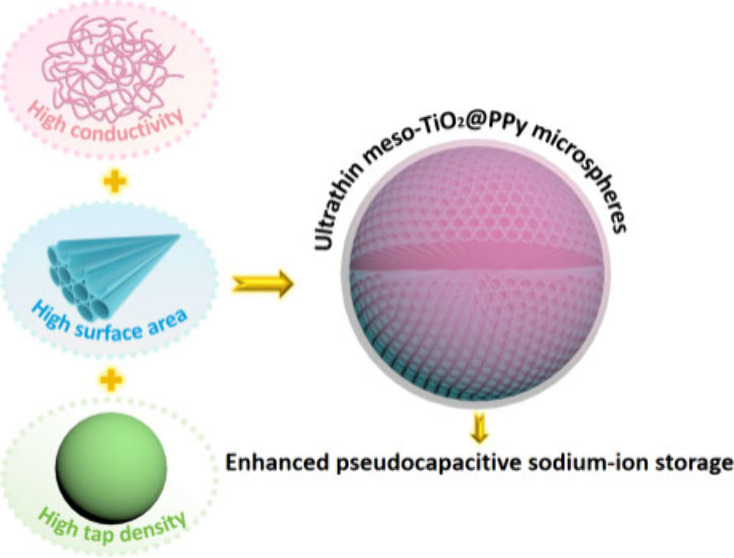

## Introduction

Electrochemical energy storage is regarded as one of the most promising research topics in the 21st century due to its application areas ranging from electric vehicles, portable electronic devices to grid systems.^1–4^ Among them, sodium-ion batteries (SIBs), characterized by the prominent natural abundance and low cost of sodium resources, are anticipated to serve as an emerging energy storage device to replace lithium-ion batteries (LIBs).^5–13^ However, the relatively higher standard electrode potential (*E*_0_(Na^+^/Na) = –2.71 V vs *E*_0_(Li^+^/Li) = −3.01 V) and larger ionic radius (Na^+^ (1.02 Å) vs Li^+^ (0.76 Å)) of sodium result in sluggish diffusion kinetics and inferior cycling stability for SIBs.^14–16^ Consequently, the development of anode materials with rapid sodium-ion diffusion pathways and extended cycling life is of paramount importance for enhancing the performance of SIBs. Currently, hard carbon materials are widely investigated as anodes for sodium-ion batteries.^17–19^ Nevertheless, their low tap density and inadequate safety limited its practical application.^20^ Transition metal oxides, such as TiO_2_ material that possesses stable crystal structure and chemical properties, have been acknowledged as a promising candidate for ideal anode materials. For instance, rutile TiO_2_ maintains good structural integrity during the intercalation and deintercalation of sodium ions, without significant volume expansion or structural collapse.^21^

Nonetheless, the employment of TiO_2_ as SIB anode still faces critical fundamental issues. First, in comparison with intercalation materials, bulk TiO_2_ that predominantly undergoes surface redox reactions has limited specific surface areas, resulting in relatively low contributions of pseudocapacitance to the total capacitance.^22^ To address this issue, the nanostructuring of TiO_2_ to maintain a high specific surface area is a common method. Ji et al. fabricated a size-tunable olive-shaped nano-TiO_2_/carbon composite and demonstrated that the particles with a size of 80 nm exhibit high-rate performance and enhanced cycling stability.^23^ Despite this, nanostructuring severely compromises the tap density of the material, thereby reducing the capacity of the battery.^22^ Constructing a mesoporous structure is an alternative and effective approach. The ample mesopores can significantly enhance the specific surface area of TiO_2_, thereby increasing the contribution of pseudocapacitance. Meanwhile, they provide efficient pathways for sodium ion diffusion, facilitate contact with the electrolyte, and do not compromise the tap density of the TiO_2_.^24–31^ Zhao et al. reported that mesoporous TiO_2_ microspheres as an anode for SIBs exhibited a high reversible capacity of 130 mAh g^–1^ at 1 A g^–1^.^22^ Similarly, Wei et al. synthesized mesoporous TiO_2_ nanosheets and employed them as anodes for SIBs, achieving remarkable performance, including a high pseudocapacitance (93.6% of total charge at 1.0 mV s^–1^) and long-term cycling stability (10,000 cycles at 10 A g^–1^).^32^ Second, TiO_2_ is a semiconductor, and its intrinsic low electrical conductivity results in low electron transfer efficiency.^28^ Previous studies have demonstrated that constructing a conductive coating lay is an effective route, such as carbon.^33–36^ However, the high-temperature carbonization process of carbon precursors typically leads to the collapse of the mesostructure and grain coarsening of TiO_2_, which are undesirable.^37–39^ In recent years, polypyrrole (PPy) has garnered attention as a coating material for various electrode materials,^40–46^ which can enhance the electrical conductivity of electrode materials without additional high-temperature treatment for sodium-ion storage.

Herein, we constructed an ultrathin PPy-coated mesoporous TiO_2_ microspheres (meso-TiO_2_@PPy) core–shell structure via a solvent evaporation-induced self-assembly method in conjunction with a chemical oxidative polymerization strategy. The product has a uniform spherical morphology in micrometer size with radially oriented TiO_2_ meso-channels and ultrathin coated PPy layer at ∼ 7 nm. The material integrates the advantages of the high specific surface area, high tap density, and rich sodium-ion diffusion channels of micrometer-sized mesoporous TiO_2_, as well as the excellent electrical conductivity provided by the ultrathin PPy layer. When applied as an anode material for SIBs, it has achieved outstanding pseudocapacitive sodium storage performances, including a high specific capacity of 160.6 mAh g^–1^ at 1 A g^–1^, remarkable rate performance (85 mAh g^–1^ at 10 A g^–1^), ultralong cyclability (80.8% retention over 2000 cycles at 1 A g^–1^), and an overwhelming capacitive contribution of 94% at 8 mV s^–1^. Density functional theory (DFT) calculations further corroborate that the meso-TiO_2_@PPy core–shell structure exhibits superior electrical conductivity and sodium storage performance, both of which facilitate the enhancement of charge–discharge specific capacity, consistent with the conclusions drawn from the experiments. Our study offers an excellent example for fabricating high-performance anode materials for SIBs and enhancing pseudocapacitive sodium storage.

## Results and Discussion

### Synthesis and Characterization

The unique structural design of meso-TiO_2_@PPy plays an important role in pseudocapacitive charge storage, encompassing several notable advantages as depicted in Figure 1a. First, the uniform and ordered mesostructure of TiO_2_, featuring internal densely stacked channels, not only significantly enhances the tap density but also simultaneously maintains a high surface area. This addresses the prevalent incompatibility between high tap density and surface accessibility in the majority of electrode materials. Second, the mesopore channels of TiO_2_ shorten the Na^+^ diffusion pathway, guaranteeing rapid Na^+^ diffusion kinetics, and the large surface area of meso-TiO_2_@PPy ensures adequate contact with the electrolyte and accelerates redox reactions. In addition, the crack structure facilitates rapid electrolyte penetration into the interior and enables transmission through the extensive internal mesoporous network. Finally, the coated ultrathin PPy layer on the surface of TiO_2_ microspheres not only does not hinder the diffusion of Na ions but also provides rich active sites for Na ions adsorption. Based on the above advantages, the meso-TiO_2_@PPy microspheres with an ultrathin PPy layer are synthesized through solvent evaporation-induced self-assembly and chemical oxidative polymerization of pyrrole methods (Figure S1). First, the mesoporous TiO_2_ microspheres (meso-TiO_2_) can be prepared through a straightforward solvent-evaporation-induced self-assembly method. Specifically, the F127/titania micelles are obtained by evaporating the tetrahydrofuran (THF) solvent at a temperature of 40 °C. Subsequently, the obtained hydrogels undergo further evaporation at 80 °C to ensure the complete removal of the THF solvent. This process leads to the formation of radial TiO_2_ meso-channels. During the above process, the synthesis of monomicelle hydrogels is the key step. The researcher should avoid rapid evaporation of THF to ensure slow hydrolysis and micellization. After annealing in air at 400 °C, the mesoporous rutile TiO_2_ microspheres are successfully formed as a result of the decomposition of organics. The yield of mesoporous TiO_2_ microspheres is 0.64 g after calcination. Afterward, an ultrathin PPy layer is coated on the surface of the mesoporous TiO_2_ through the chemical oxidation polymerization of the pyrrole (Py) in an ice bath (Figure S1). This final step results in the ultimate meso-TiO_2_@PPy composite.

**Figure 1:**
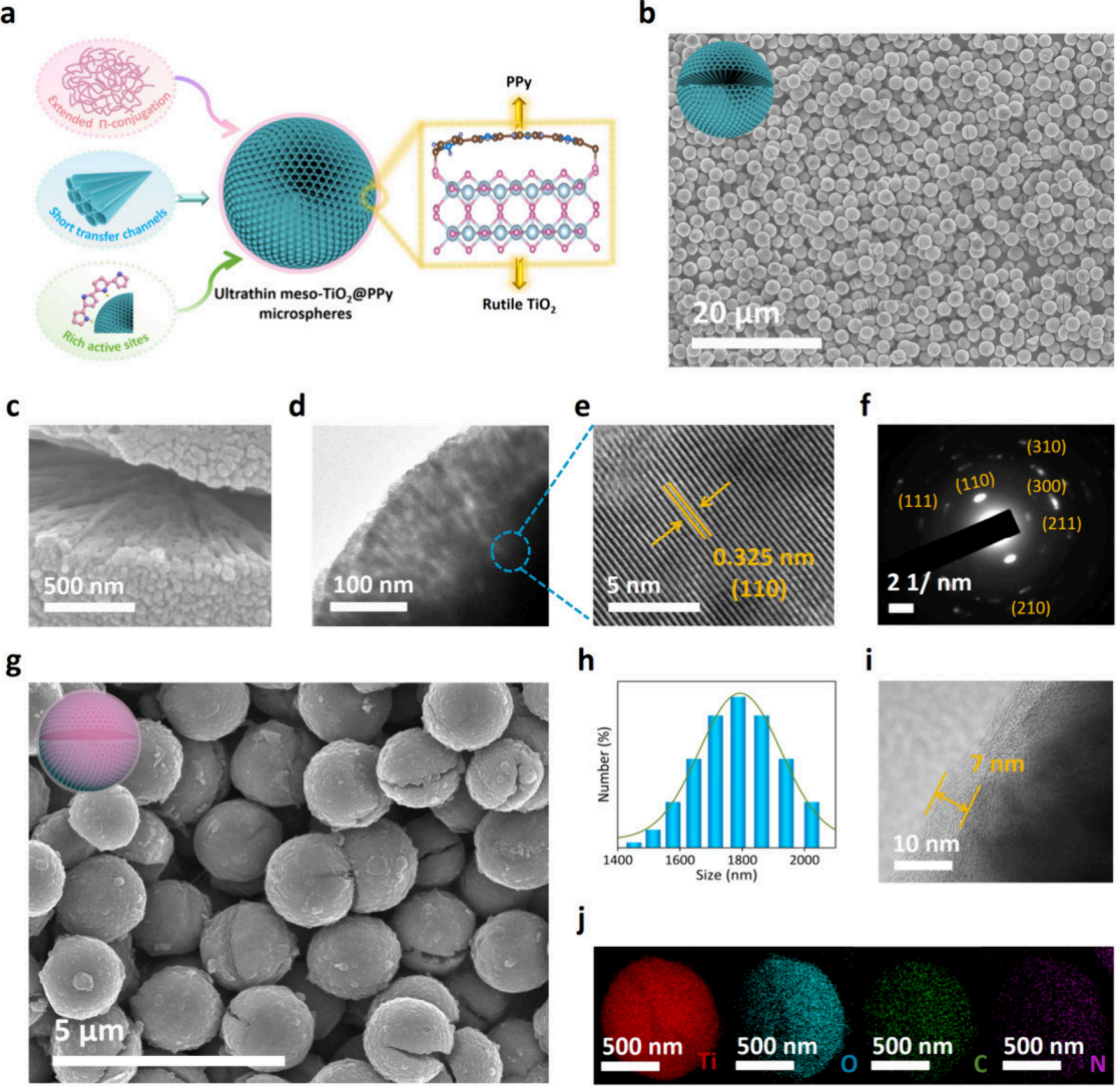
Material design and synthesis of the mesoporous composite. (a) Schematic illustration for the material design of meso-TiO_2_@PPy microspheres. (b, c) SEM images, (d) TEM image, (e, f) HRTEM image, and SAED pattern of the uniform meso-TiO_2_ microspheres. (g) SEM image, (h) Size distribution, (i) TEM image of the meso-TiO_2_@PPy-7 composite. (j) EDX elemental mappings of meso-TiO_2_@PPy-7, including Ti, O, C, and N elements.

The scanning electron microscopy (SEM) images display that the obtained meso-TiO_2_ has a uniform and monodispersed cracked spherical morphology (Figure 1b and Figure S2), and close inspections further display that the mesoporous frameworks composed of TiO_2_ nanorods aligned from the core to the exterior are discernible within the obtained microspheres (Figure 1c,d). The high-resolution transmission electron microscopy (HRTEM) image indicates a parallel lattice spacing of 0.325 nm, which corresponds to the (110) plane of rutile TiO_2_ (Figure 1e and Figure S3). In addition, the selected-area electron diffraction (SAED) pattern of meso-TiO_2_ shows a set of diffraction rings that were indexed to the (110), (200), (111), (210), (211), (310) and (220) planes of the rutile phase, revealing the formation of polycrystalline rutile phase (Figure 1f). After depositing the PPy coating, a uniform layer envelops the meso-TiO_2_ microspheres, with an average diameter of approximately 1.8 μm, indicating a consistent coating process (see Figure 1g,h and Figure S4). As shown in Figure 1i, the thickness of the PPy layer is about 7 nm (marked as meso-TiO_2_@PPy-7). The PPy coating thickness is easily adjusted to be 25 nm (marked as meso-TiO_2_@PPy-25) and 50 nm (marked as meso-TiO_2_@PPy-50) by changing the amount of pyrrole in the solution (Figure S5). Elemental mapping images reveal that a homogeneous distribution of the Ti, O, C, and N elements in the meso-TiO_2_@PPy-7 material, which indicates PPy coating is uniform on the surface of meso-TiO_2_ (Figure 1j). After the PPy coating, the zeta potential of the meso-TiO_2_ is significantly improved from −44.5 to +6.4 mV at pH = 7.0 (Figure S6).

As shown in Figure 2a, the electron paramagnetic resonance (EPR) results demonstrate that the meso-TiO_2_ has oxygen vacancies, these oxygen vacancies play a pivotal role in enhancing charge transfer at the interface, thereby markedly improving both conductivity and the contribution of pseudocapacitance. In addition, an appropriate amount of oxygen vacancies can provide additional intercalation sites for sodium ions, thereby enhancing the sodium storage capacity. The X-ray powder diffraction (XRD) patterns of both the meso-TiO_2_ and meso-TiO_2_@PPy-7 exhibit well-defined diffraction peaks within the 2θ range from 20° to 80°, which can be indexed with the rutile phase (*I*4_1_/*amd*). Notably, the meso-TiO_2_@PPy-7 shows a slightly declined intensity due to being covered by the PPy layer (Figure 2b). From N_2_ adsorption–desorption isotherms, the meso-TiO_2_ and meso-TiO_2_@PPy-7 both exhibit a typical IV adsorption/desorption isotherm at *P/P*_*0*_ = 0.4–0.9, revealing the uniform and well-defined mesoporosity (Figure 2c). As calculated by the Brunauer–Emmett–Teller (BET) method, the meso-TiO_2_@PPy-7 indicates a higher specific surface area (144 m^2^ g^–1^) than that of meso-TiO_2_ (119 m^2^ g^–1^), which is associated with the loose structure of PPy. The meso-TiO_2_@PPy-7 exhibits a narrow pore size distribution at about 3.2 nm (Figure 2d), and the pore volume is calculated to be a relevant high value of ∼ 0.41 cm^3^ g^–1^ (Table S1). These differences in specific surface area and pore volume would drastically benefit the sodium ion storage behavior.

**Figure 2:**
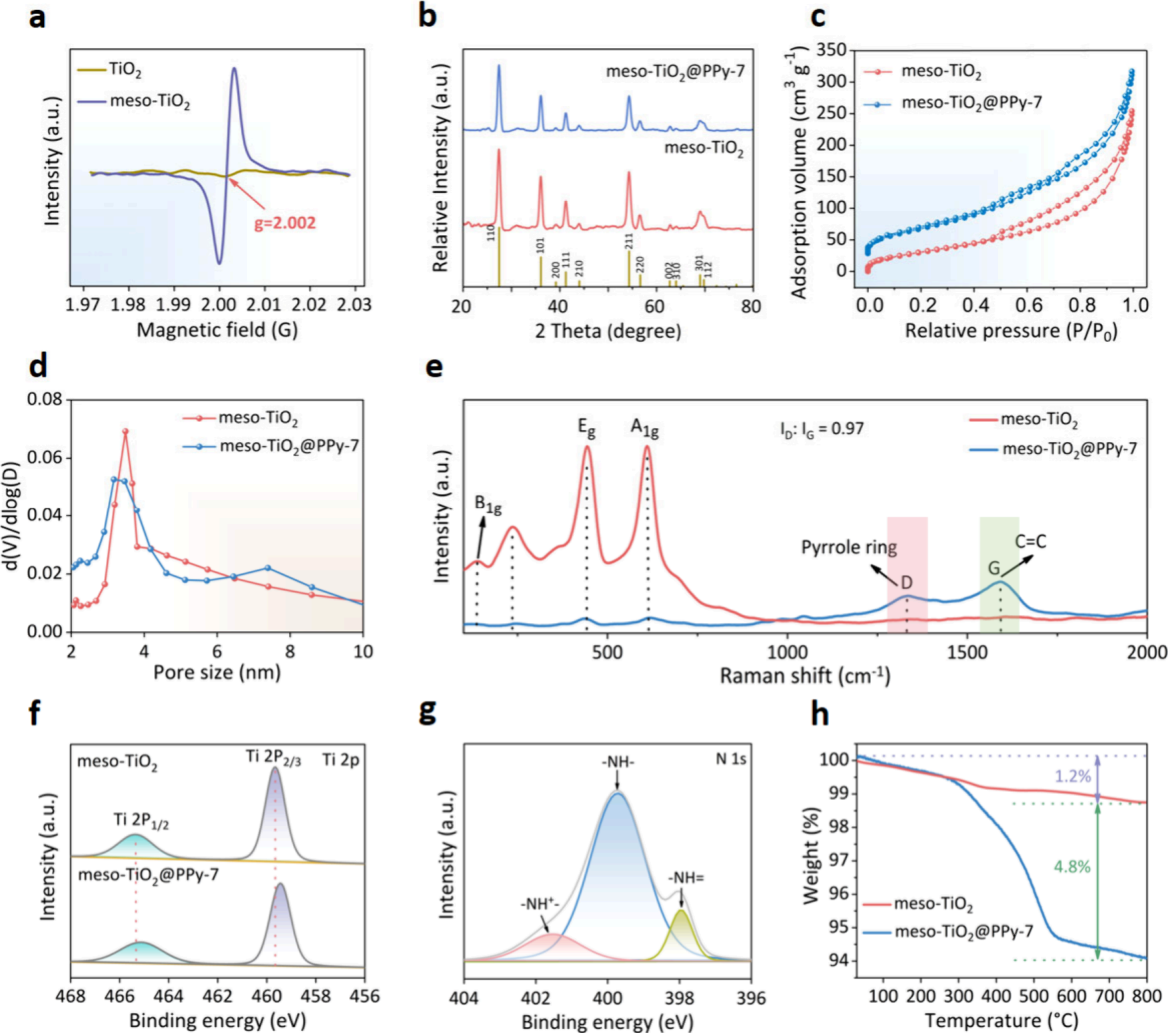
Characterizations of the designed mesoporous microspheres. (a) EPR spectra of the meso-TiO_2_ and TiO_2_ nanoparticles. (b) XRD patterns, (c) N_2_ adsorption–desorption isotherms, (d) Pore size distribution curves, (e) Raman spectra of the bare meso-TiO_2_ and meso-TiO_2_@PPy-7 microspheres. (f) High-resolution Ti 2p XPS spectra for the meso-TiO_2_ and meso-TiO_2_@PPy-7. (g) High-resolution N 1s XPS spectra for the meso-TiO_2_@PPy-7. (h) TGA curves of the meso-TiO_2_ and meso-TiO_2_@PPy-7 microspheres.

Raman spectroscopy shows that the meso-TiO_2_@PPy-7 inherits the characteristic signals of both meso-TiO_2_ and PPy (Figure 2e). The bands at 137, 437, and 621 cm^–1^ correspond to the typical B_1g_, E_g_, and A_1g_ vibrational modes of the Ti–O bond, while the band at 239 cm^–1^ is ascribed to the lattice disorder of rutile.^47^ The bands at 1337 and 1592 cm^–1^ are typical D and G lines of PPy layers. A large I_D_/I_G_ value (0.97) in meso-TiO_2_@PPy-7 indicates that the PPy layer is amorphous. From the X-ray photoelectron spectroscopy (XPS) spectrum, the characteristic peaks of Ti, O, N, and C elements in meso-TiO_2_@PPy-7 are observed (Figure S7a). In the Ti 2p XPS spectrum, the Ti 2p peak shifts to lower energy after coating PPy, suggesting the presence of a bonding interaction and the establishment of a heterostructure between meso-TiO_2_ and PPy (Figure 2f). The shifts in the C 1s and O 1s signals disclose that the O–C bond between TiO_2_ and PPy is formed,^48,49^ further implying the successful fabrication of mesoporous heterojunction (Figure S7b,c). The N 1s high-resolution spectrum fits into two peaks, in which the features at 397.2 and 399.7 eV are consistent with the imine-like (=N−) atoms and pyrrole nitrogen (−NH−), respectively (Figure 2g). Additionally, the peak at 401.5 eV suggests the presence of charged nitrogen species (−NH^+^−).^50^ Besides, the PPy content in the meso-TiO_2_@PPy-7 microspheres was examined by thermogravimetric analysis (TGA), showing the weight loss of 4.8% derived from the PPy content (Figure 2h).

### Electrochemical Performances

To verify the possible use of such a designed mesoporous composite in electrochemical performance, the meso-TiO_2_ and meso-TiO_2_@PPy-7 materials as SIB anodes were measured and compared. In the Electrochemical Impedance Spectroscopy (EIS) spectra (Figure 3a and Figure S8), a smaller semicircle in the high-to-medium frequency can be observed for the meso-TiO_2_@PPy-7 electrode than for the meso-TiO_2_, showing that the meso-TiO_2_@PPy-7 has a smaller resistance of 30 Ω than that of bare meso-TiO_2_ (43 Ω), indicating the increased electrical conductivity after PPy coating. In addition, a voltage drop of 0.056 V for the meso-TiO_2_@PPy-7 is much smaller than that for the meso-TiO_2_ (0.210 V) (Figure 3b), which indicates PPy coating provides an effective electron and charge conduction pathway. Compared with the meso-TiO_2_, the meso-TiO_2_@PPy-7 anode exhibits high discharge/charge capacity (159.1/160.6 mAh g^–1^) at a current density of 1 A g^–1^ (Figure 3c). The corresponding initial Coulombic efficiencies (ICE) are calculated to be as high as 82%. As summarized in Table S2, although the cyclic stability of the meso-TiO_2_@PPy-7 needs to be further improved, its capacity is higher than other TiO_2_-based materials at a current density of 1 A g^–1^. Besides, the meso-TiO_2_@PPy-7 exhibits higher specific capacity at various current densities than the meso-TiO_2_ (Figure 3d). In addition to the remarkable rate performance, the meso-TiO_2_@PPy-7 anode also displays a very high specific capacity of 160.6 mAh g^–1^ with a high retention of 80.8% after 2000 cycles, which is much higher than meso-TiO_2_ (72.2 mAh g^–1^) (Figure 3e). Because the active materials need several cycles to fully participate in the reaction, and the electrolyte also need to gradually penetrate the electrode material to form a stable interface layer, this causes the capacity of the meso-TiO_2_@PPy ascend gradually at the beginning. It is worth noting that the nanostructure and crystalline phase of the meso-TiO_2_@PPy-7 are well maintained even after 2000 cycles at 1 A g^–1^, which indicates the stability of the material (Figure S9). The enhanced electrical conductivity, rate performance, and capacity of meso-TiO_2_@PPy-7 are ascribable to the presence of PPy coating.

**Figure 3:**
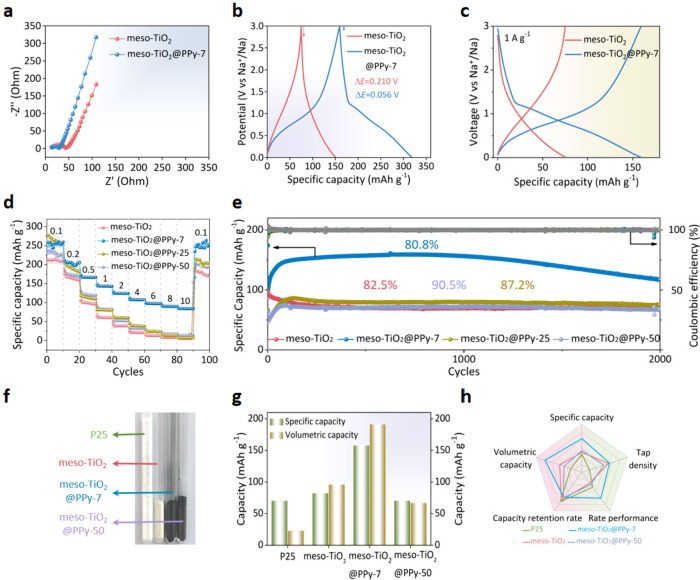
Electrochemical measurements. (a) EIS curves of the meso-TiO_2_ and meso-TiO_2_@PPy-7 electrodes. (b) GCD profiles of meso-TiO_2_ and meso-TiO_2_@PPy-7 electrodes at 1 A g^–1^. (c) Charge–discharge profiles at cycles of meso-TiO_2_ and meso-TiO_2_@PPy-7 electrodes at 1 A g^–1^. (d) Rate performances of the meso-TiO_2_, meso-TiO_2_@PPy-7, meso-TiO_2_@PPy-25 and meso-TiO_2_@PPy-50 anodes ramping from 0.1 to 10 A g^–1^ and back to 0.1 A g^–1^. (e) Cycling stability of the electrodes during 2000 cycles at 1 A g^–1^. (f) Photographic image of the four samples. All vials contain 200 mg of tightly packed powders. (g) Gravimetric and volumetric capacities of the four electrodes at 1 A g^–1^. (h) Comparison of electrochemical properties of different electrodes.

We further explored the effect of PPy layer thickness on the sodium storage performance of meso-TiO_2_. As shown in Figure 3d,e, the rate performance of the meso-TiO_2_@PPy-25 and meso-TiO_2_@PPy-50 is lower than that of the meso-TiO_2_@PPy-7. In addition, the meso-TiO_2_@PPy-25 and meso-TiO_2_@PPy-50 also exhibit lower specific capacities of 89.5 and 74.2 mAh g^–1^ at 1 A g^–1^. As the PPy thickness increases, the meso-TiO_2_@PPy-25 and meso-TiO_2_@PPy-50 anodes exhibit low discharge/charge capacity at a current density of 1 A g^–1^ and high voltage drop (Figure S10). These responses suggest that the excessively thick PPy coating prevents charge transportation within the internal mesoporous frameworks. To validate the optimal thickness of PPy layer on the surface of mesoporous TiO_2_, we further evaluated the performance of the meso-TiO_2_@PPy-4 sample, featuring a thinner PPy layer of ∼ 4 nm (Figure S11a,b). The specific capacity of meso-TiO_2_@PPy-4 was 101.3 mAh g^–1^ (Figure S11c), significantly lower than that of meso-TiO_2_@PPy-7 (160.6 mAh g^–1^). This response is attributable to the insufficient PPy layer for complete surface coverage to form a substantial solid electrolyte interphase (SEI) layer, resulting in undesirable conductivity and sluggish sodium ion diffusion kinetics. It should be noted that the capacity of the meso-TiO_2_@PPy exhibits an initial gradual increase, which is attributed to the PPy coating hindering electrolyte wetting kinetics for a stable SEI layer. Also, a thicker PPy layer leads to decreased capacity, mainly because an excessive coating layer would lead to a thick SEI layer during the initial sodiation process to impede sodium-ion diffusion while the effective access to the electrochemically active mesoporous TiO_2_ is diminished at the meantime.

Furthermore, to demonstrate the densely packed design, we measured the volumetric capacity of such mesoporous composite microspheres. After incorporating the PPy layer, the tap density of meso-TiO_2_@PPy-7 (1.21 g cm^–3^) is comparable to that of meso-TiO_2_ (1.17 g cm^–3^), and much higher than that of commercial P25, indicating the high spatial utilization with well-preserved high-surface-area structure (Figure 3f and Figure S12). In turn, this feature endows the meso-TiO_2_@PPy-7 with a high-volume capacity of 191.8 mAh cm^–3^, which is almost twice that of meso-TiO_2_ (Figure 3g). All these electrochemical performances of meso-TiO_2_@PPy-7 are outstanding in terms of capacity retention rate, rate performance at 10 A g^–1^, tap density, specific capacity, volumetric capacity (Figure 3h), highlighting the significance of the PPy layer and mesoporous design in promoting the sodium storage performance of TiO_2_.

### Dynamic Analysis

To deeply understand the sodium storage behavior of the TiO_2_-based electrode materials, the cyclic voltammetry (CV) curves were tested on the meso-TiO_2_ and meso-TiO_2_@PPy-7 at the scan rates from 0.2 to 8 mV s^–1^ (Figure 4a,b). At a sweep rate of 0.2 mV s^–1^, a pair of redox peaks located at 0.8 and 0.7 V (vs Na/Na^+^) corresponds to the Ti^3+^/Ti^4+^ redox couple, in agreement with charge–discharge results. CV curves show similar broad peaks with increasing currents with the rise of sweep rates. The *b*-values were further calculated by plotting log­(*i*) against log­(*v*) to analyze the charge-storage mechanism. The *b*-values for cathodic and anodic peaks of the meso-TiO_2_@PPy-7 are calculated to be 0.99 and 0.95, which are higher than those of meso-TiO_2_ (Figure 4c,d), demonstrating the fast electrochemical kinetics governed by pseudocapacitive behavior. Furthermore, the charge storage contribution from pseudocapacitance was also differentiated quantitatively. An overwhelming proportion of 94% for the meso-TiO_2_@PPy-7 and 77% for the meso-TiO_2_ of the total charge is contributed from the capacitive process at 8 mV s^–1^, suggesting the capacitive dominated process (Figure 4e,f). More ions can be diffused into the electrode with the increase of sweep rate, leading to the increased capacitive contribution (Figure 4g,h). These results validate that the integrated conductive PPy layer and uniform TiO_2_ mesostructure favor to realization of excellent pseudocapacitance properties of the meso-TiO_2_@PPy-7. Furthermore, the Nyquist plots of meso-TiO_2_@PPy-7 show a small semicircle at high frequency, and become linear at low frequency as the temperature rises due to the diffusion of Na^+^ (Figure S13), indicating the improved stability and diffusion kinetics of sodium ions.

**Figure 4:**
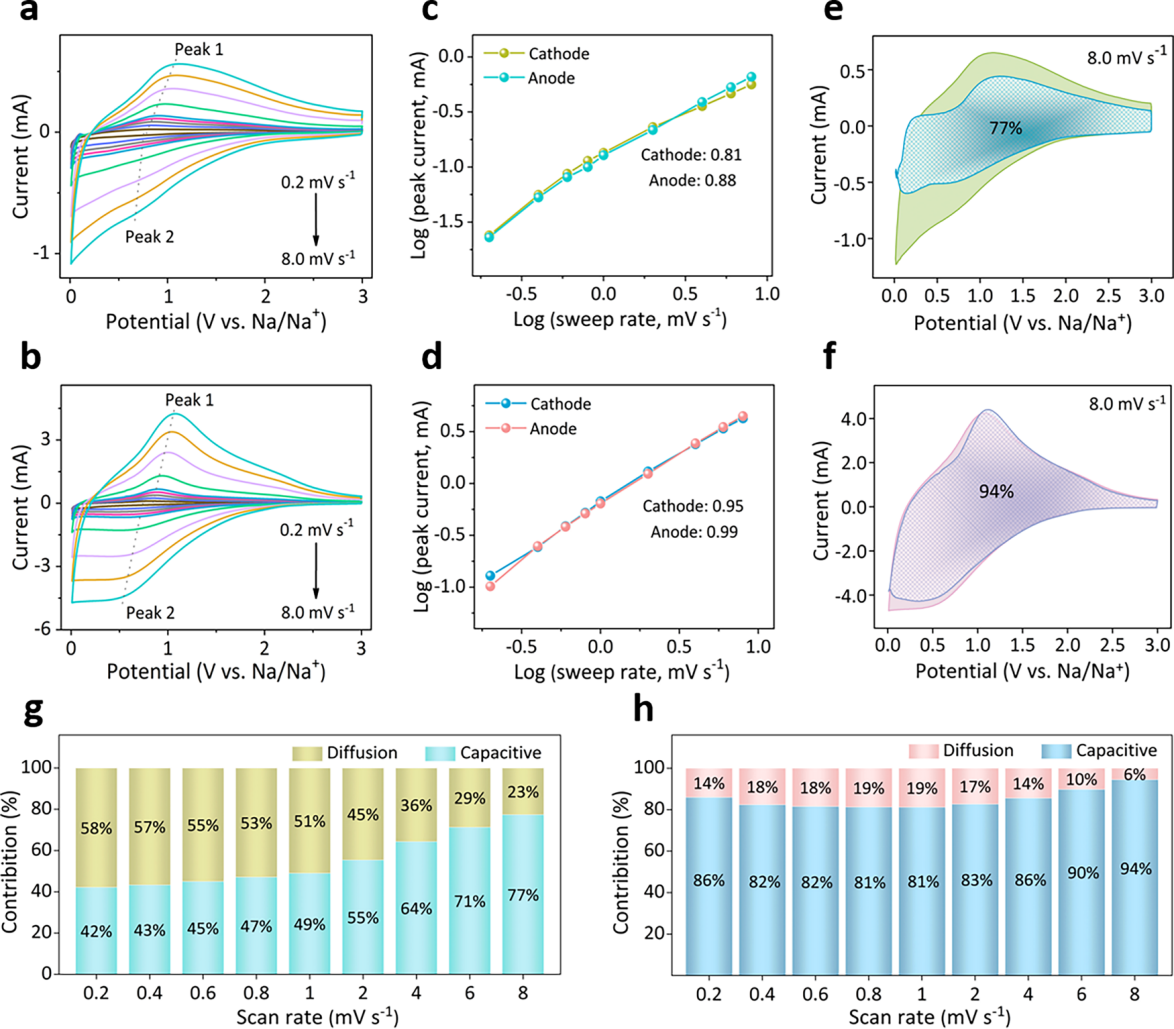
Dynamic analyses of meso-TiO_2_ and meso-TiO_2_@PPy-7. CV curves of (a) meso-TiO_2_ and (b) meso-TiO_2_@PPy-7. The *b*-values of (c) meso-TiO_2_ and (d) meso-TiO_2_@PPy-7. The pseudocapacitive contribution ratios of (e) meso-TiO_2_ and (f) meso-TiO_2_@PPy-7 at 8 mV s^–1^. Pseudocapacitive contribution of (g) meso-TiO_2_ and (h) meso-TiO_2_@PPy-7 at different scan rates.

### Theoretical Calculation

To gain fundamental insights into the effect of PPy coating on the sodium storage performance, the density functional theory (DFT) calculations were performed at the molecular and electronic levels. As shown in Figure 5a, the Ti–O–C bonds are formed between the TiO_2_ and PPy molecules, and the binding energy is about −0.20 eV, suggesting that the meso-TiO_2_@PPy is very stable. Figure 5b depicts the differential charge density distribution of meso-TiO_2_@PPy, the number of Bader charges shifted from the PPy to TiO_2_ is about 1.2e, revealing significant charge transfer between PPy and TiO_2_. Furthermore, the meso-TiO_2_@PPy shows a narrower band gap (0.8 eV) than that of the pure meso-TiO_2_ (1.7 eV) and PPy (1.4 eV), indicating improved electronic conductivity (Figure 5c–e, Figure S14). The enhanced electrical conductivity of meso-TiO_2_@PPy is attributed to the production of a new state and exhibiting more occupations around the Fermi level (Figure S15). To investigate the origin of the meso-TiO_2_@PPy with high specific capacity, the Na^+^ adsorption energy (Δ*E*_abs_) of pure meso-TiO_2_ and meso-TiO_2_@PPy models was calculated, respectively. The meso-TiO_2_@PPy exhibits a lower adsorption energy of Na^+^ (Δ*E*_abs_ = −0.96 eV) than the meso-TiO_2_ (Δ*E*_abs_ = 1.03 eV) (Figure 5f and Figure S16), demonstrating the facilitated sodium charge storage.

**Figure 5:**
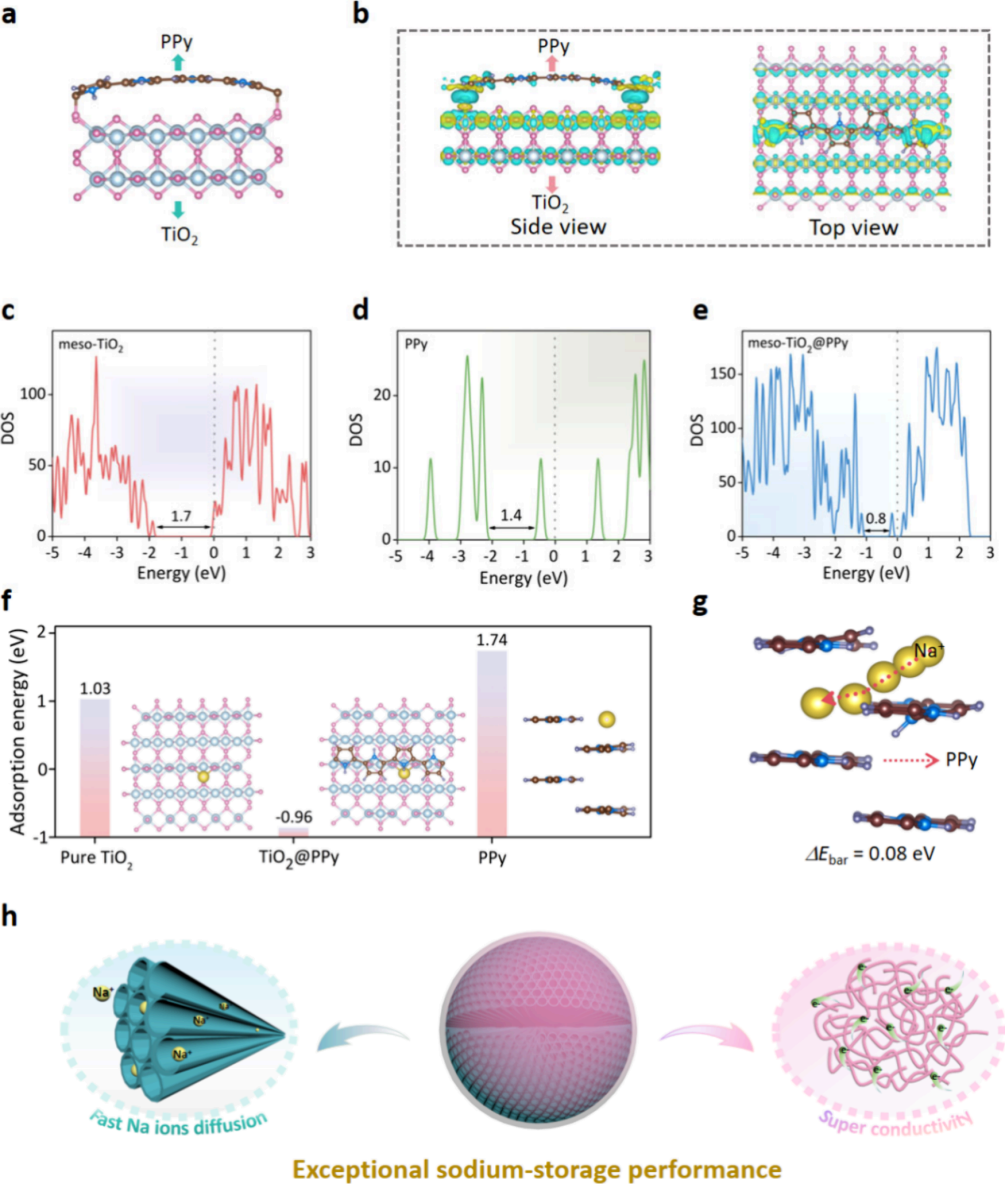
Theoretical calculations. (a) The structure model of the meso-TiO_2_@PPy. (b) Differential charge density distribution of TiO_2_@PPy (yellow regions, increased electron density; blue regions, decreased electron density). (c–e) The DOS of meso-TiO_2_, PPy, and meso-TiO_2_@PPy. (f) The adsorption energy of Na^+^ on the surface of meso-TiO_2_, meso-TiO_2_@PPy, and PPy frameworks. (g) The energy barrier of Na^+^ through PPy layers. (h) Scheme of the core–shell mesoporous electrode for enhanced sodium storage. Color code: Ti (cyan), O (pink), H (purple), N (dark blue), C (brown), Na (yellow).

In addition, we used the 4-layer PPy model to simulate the meso-TiO_2_@PPy with a thick PPy layer to investigate the thickness effect. As shown in Figure 5f and Figure S17, the PPy model exhibits a higher adsorption energy of Na^+^ (Δ*E*_abs_ = 1.74 eV) than both meso-TiO_2_ and meso-TiO_2_@PPy, indicating that the thick PPy coating is not conducive to the storage of sodium. Furthermore, the DFT study shows that the Na^+^ needs to overcome an energy barrier of 0.08 eV across each PPy layer (Figure 5g), making it difficult for Na^+^ to move through the thick PPy layer. Although the thick PPy coating has enhanced the conductivity (Figure S18), the high adsorption energy of Na^+^ results in poor electrochemical performance. Thus, the excellent electrochemical performance of ultrathin PPy-coated mesoporous TiO_2_ is mainly associated with its unique core–shell structure, as illustrated in Figure 5h. These heightened interests are attributed to the numerous advantages of PPy as a coating material. The mesopore channels of TiO_2_ shorten the Na^+^ diffusion pathway, ensuring the fast Na^+^ kinetics and resulting in superior rate performance and fast pseudocapacitive sodium-ion storage. The densely packed microspheres on a micrometer scale offer both improved tap density and high surface area, leading to both high gravimetric and volumetric capacities. The ultrathin PPy layer offers excellent conductive pathways, thereby diminishing interfacial resistance and enhancing the overall energy and power densities of the SIB battery. Moreover, the coating also serves as a protective layer for TiO_2_, shielding it from the influence of the external environment.

## Conclusion

In summary, a type of 3D mesoporous TiO_2_@PPy composite material is designed and synthesized through a solvent evaporation-induced self-assembly method in conjunction with a chemical oxidative polymerization to promote the low electronic conductivity and sluggish diffusion kinetics of TiO_2_. The obtained meso-TiO_2_@PPy microspheres possess a uniform spherical microstructure with radially aligned TiO_2_ meso-channels, an ultrathin coated PPy layer at ∼ 7 nm, as well as good mesoporosity (a high surface area of 144 m^2^ g^–1^, mean pore size at 3.2 nm). The unique structural features of mesoporous TiO_2_ coupled with PPy layer not only increase the charge diffusion internally and externally but also provide rich active sites for sodium ions storage. As an anode material for SIBs, the meso-TiO_2_@PPy-7 demonstrates excellent rate performance and a higher specific capacity of 160.6 mAh g^–1^ up to 2000 cycles at a current density of 1.0 A g^–1^, which is a dramatic improvement compared with meso-TiO_2_ (72.2 mAh g^–1^). Such structural design also provides a highly enhanced tap density (1.21 g cm^–3^) and volumetric capacity (191.8 mAh cm^–3^), along with a dominant pseudocapacitive contribution of 94% at 8 mV s^–1^. Moreover, DFT calculations and experimental studies reveal that a relatively thick PPy layer is not conducive to Na^+^ diffusion, thus decreasing the specific capacity. Our work is envisaged to offer insights for designing integrated mesoporous materials with rapid pseudocapacitive sodium storage and high volumetric density.

## Supplementary Material



## Notes

Additional experimental details for the calculations, characterizations of materials, and electrochemical measurements (PDF)
