# Supplementary material for: Precisely Integrated Mesoporous Anode Enabling Fast Pseudocapacitive Sodium-Ion Storage
Source: ACS Cent Sci. 2025 Aug 18;11(10):1892–901. doi: 10.1021/acscentsci.5c00616 (PMC12550626; doi:10.1021/acscentsci.5c00616)
Supplement: Supplementary file 1 [file oc5c00616_si_001.pdf]

Supporting Information

Precisely Integrated Mesoporous Anode Enabling Fast Pseudocapacitive Sodium-Ion Storage

Shuang Li,[1] Jiecheng Chen,[1] Xin Miao,[1] Xu Wen,[1] You Zhou,[1] Bingxian Chu,[2] Wendi Wang,[1] Yanyan Yu,[1] Ziyang Guo,[1]* Kun Lan[1]*

[1]College of Energy Materials and Chemistry, College of Chemistry and Chemical Engineering, Inner Mongolia University, Hohhot 010021, P. R. China.

[2]Department of Materials Science and Engineering, Southern University of Science and Technology, Shenzhen 518055, P. R. China.

*Email: k_lan@imu.edu.cn; zyguo@imu.edu.cn

Experimental procedures

Materials

Pluronic triblock copolymer poly-(propylene oxide)-*block*-poly(ethylene oxide)-*block*-poly(propylene oxide) (F127, M_w = 12600, $EO_{106}PO_{70}EO_{106}$) was purchased from Acros Corp. Tetrabutyl titanate (TBOT, 99.5%), pyrrole (Py, 98.0%) and ammonium persulfate (APS, 99.99%) were purchased from Sigma-Aldrich Corp. Tetrahydrofuran (THF), acetic acid, and hydrochloric acid (HCl, 36-38 wt%) were purchased from Sinopharm Chemical Reagent Co., Ltd. (China). All reagents were purchased from commercial sources and used without further purification. Deionized water was used for all experiments.

Synthesis of meso-TiO_2 microspheres

The meso-TiO_2 microspheres were synthesized according to the approach of ref.[1] Specifically, 1.6 g of triblock copolymer F127, 2.6 mL of acetic acid, and 2.0 mL of concentrated HCl were added into 30.0 mL of THF in sequence to form a transparent solution under stirring (stirring rate of 400 r min^{-1}). The mixture was stirred for 5 min to form a clear solution. Then, 3.4 mL of TBOT was added to the mixed solution. After stirring for another 5 min, a red solution was formed and then transferred into two volumetric flasks (30 mm × 30 mm). After that, it was kept in an oven at 40 °C for 15 h to preferentially evaporate THF, then at 80 °C for another 10 h. Subsequently, the white precipitates were washed several times with ethanol and collected by centrifugation, and dried in an oven. Finally, the meso-TiO_2 microspheres were obtained after calcination under air at 400 °C for 3 h (with a heating rate of 2 °C min^{-1}).

Synthesis of meso-TiO_2@PPy microspheres

The PPy shells were coated on the meso-TiO_2 surface according to the method of ref.[2] Specifically, 0.25 g of the obtained meso-TiO_2 microspheres were dispersed in 240 mL of deionized water and stirred for 12 h. Then, 15.0 μL of pyrrole, 2.0 mL of 1 mol L^{-1} HCl, and 20.0 mL of 0.01 mol L^{-1} APS aqueous solution were successively added into the above solution and stirred (stirring rate of 600 r min^{-1}) for 6 h in an ice-water bath. Finally, the black precipitates were washed several times with deionized water and collected by centrifugation, and dried in an oven. The different PPy shell thicknesses were obtained through a similar process, except for varying the amount of pyrrole.

DFT calculations

DFT calculations were performed by the planewave pseudopotential method implemented in the Vienna Ab-initio Simulation Package (VASP).[3,4] The generalized gradient approximation of the Perdew-Burke-Ernzerh of exchange−correlation function was depicted through a plane wave with an energy cutoff of 400 eV to ensure the precision of the calculations.[5] The pristine rutile TiO_2 with 4 × 2 × 1 unit cells (40 Ti atoms and 80 O atoms) was used for the calculations, and the foremost growth surface (110) was used to establish the supercell. For calculations of geometries, the integrations over the Brillouin zone were performed with a 1 × 1 × 1 special k-point mesh. The convergence criterion for the electronic self-consistent cycle was controlled at 0.01 eV percell. The geometry optimization was stopped when the forces on all unconstrained atoms were less than 0.05 eV $Å^{-1}$.

Material characterizations

The morphology and structure of samples were observed by field-emission scanning electron microscopy (FESEM, Regulus 8100). The mesoporous structure and PPy layer of samples were observed by transmission electron microscopy (TEM) and energy dispersive X-ray spectroscopy (EDX) with a Tecnai F20 transmission electron microscope (200 kV). The composition and structure of the samples were gained

by powder X-ray diffraction (XRD) using a PANalytical Empyrean diffractometer with Cu Kα radiation (k = 1.5406 Å). X-ray photoelectron spectra (XPS) were gathered using a Thermo Scientific ESCALAB Xi+ using Al Kα as the excitation source. The mesoporosity of the samples was measured using N_2 adsorption-desorption isotherms at 77 K. The Brunauer-Emmett-Teller (BET) method was used to calculate surface areas, and the Barrett-Joyner-Halenda (BJH) model was used to calculate pore volumes and pore sizes from adsorption branches. The thermogravimetric analysis (TGA) tests were conducted using the PerkinElmer TGA 4000 equipment from 30 to 800 °C with a heating rate of 10 °C min^{-1}. The electron paramagnetic resonance (EPR) measurements were recorded using a EMXplus spectrometer at room temperature. Raman microscopy at an excitation wavelength of 514 nm and Fourier-transform infrared spectrophotometry (FTIR). Zeta potential measurements were performed using a NanoBrook 90plus PALS.

Electrochemical measurements

The sodium-storage properties of all the samples were characterized by fabricating CR2032 coin-type semicells in an Ar glove box. The electrode was fabricated via mixing active material, acetylene black, the CMC, and SBR binder in an 8:1:0.5:0.5 by weight ratio of deionized water and ethanol solvent. The diameter of the sodium anode of the button battery was 12 mm, and the amount of electrolyte used was 70 μL. Generally, the active substance has a mass load of ~0.8-1.2 mg cm^{-2}. The slurry was cast onto coated carbon aluminum foil and dried at 120 °C in a vacuum oven for 12 h. The electrolyte contains 1.0 M $NaPF_6$ in diglyme. The resulting 2032 coin-type cells are assembled in a glove box using pure sodium as the anode, a glass fiber separator (Whatman, GF/A) saturated, and a micro-porous membrane as the separator. The CV measurements were carried out on a (Autolab: CH Metrohm) electrochemical workstation. The responses of the test cycle performance and rated capacity were recorded by the LAND-BT2013A measurement system at 25 °C. Galvanostatic charge and discharge (LANHE CT3002A) tests were carried out in the voltage range of 0.01~3.0 V. The rapid capacitive dynamics of TiO_2 material were investigated using in situ electrochemical impedance spectroscopy (EIS) in the frequency range of 10^{-2} to 10^5 Hz.

Supplementary Text

1. Calculating tap density of compressed electrodes

The tapped density (ρ) is calculated using the formula：

$$\rho = m/V$$

where m denotes the mass of the material, and V represents the volume occupied by the material after compaction.

2. Calculating volumetric capacity

The volumetric capacity (C_V) is calculated using the formula：

$$C_V = \rho \times C$$

where ρ denotes the tapped density of the material, and C represents the specific capacity.

3. Analyses of pseudocapacitive diffusion dynamics

Generally, the relationship between peak current (i, mA) and scan rate (v, mV s^{-1}) can be described as follows.[6,7]

$$i = av^b$$

where a and b are variable constants. The charge storage mechanism is closely related to the b value. If b = 0.5, the diffusion process dominates the electrochemical process, while if b = 1.0, pseudo-capacitance behavior controls the reaction. As shown in Figure 4, by linear fitting the plot of

log (i) vs. log (ν), all the calculated b values of the reduction and oxidation peaks are close to 1.0, indicating that the sample exhibits typical pseudocapacitance behaviors. Quantitative analysis:

$$i(V) = k_1\nu + k_2\nu^{1/2}$$

demonstrates that the pseudocapacitance contribution ratios of the sample play an increasingly important role as the scanning rate increases.

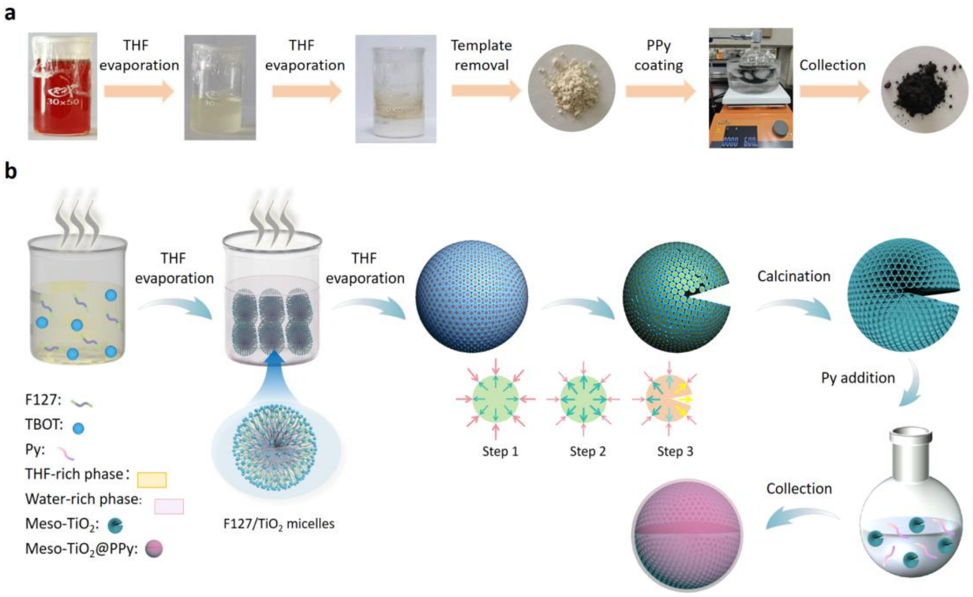

Figure S1. Synthetic procedures of the meso-TiO_2@PPy microspheres. (a) Photographic images and (b) Schematic illustration for the synthesis of the meso-TiO_2@PPy microspheres at different stages. During the synthesis process of mesoporous TiO_2, the acidic THF solution was first evaporated at 45 °C to form spherical mono-micelles. Then, evaporation continued at 80 °C, and these spherical mono-micelles

tend to aggregate to form large, dense microspheres to reduce interface tension energy. The formed large particles contain solvents, including high-boiling *n*-butanol from tetrabutyl titanate (TBOT) hydrolysis, as well as a spot of residual THF and water (state 1). With continuous hydrolysis and condensation, the amount of n-butanol and water gradually increases, resulting in an enhancement of the internal vapor pressure (state 2). The vapor pressure inside the weighing bottle ultimately exceeds the exterior hydrothermal pressure, and the solvents inside prefer to evaporate from partial regions, resulting in the formation of cracks on the surface of the microspheres (state 3). In addition, the increased temperature can cause intensive hydrolysis and a resultant higher vapor pressure inside microspheres. An explosive evaporation further occurs upon overwhelming internal vapor pressure, forming destroyed microspheres.

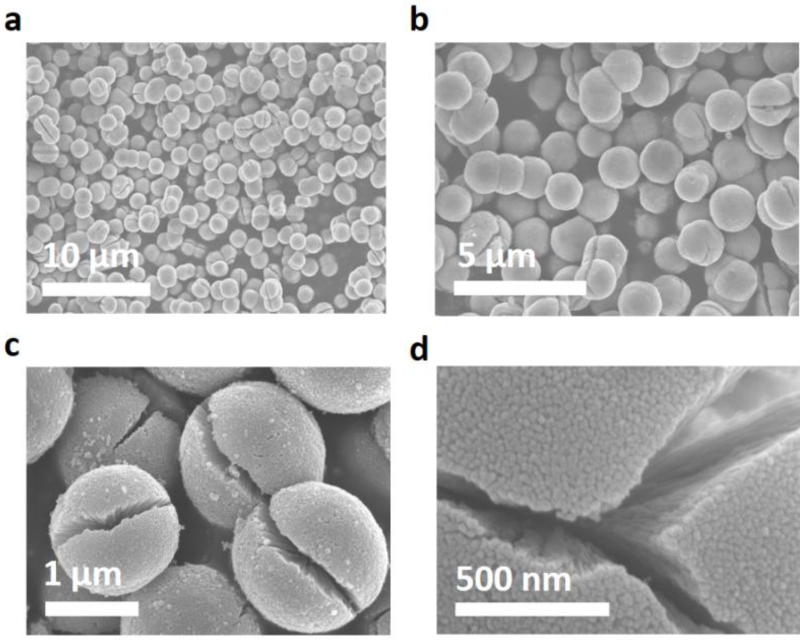

Figure S2. (a-d) FESEM images with different magnifications and structure of the meso-TiO_2 microspheres after calcination in air at 400 °C.

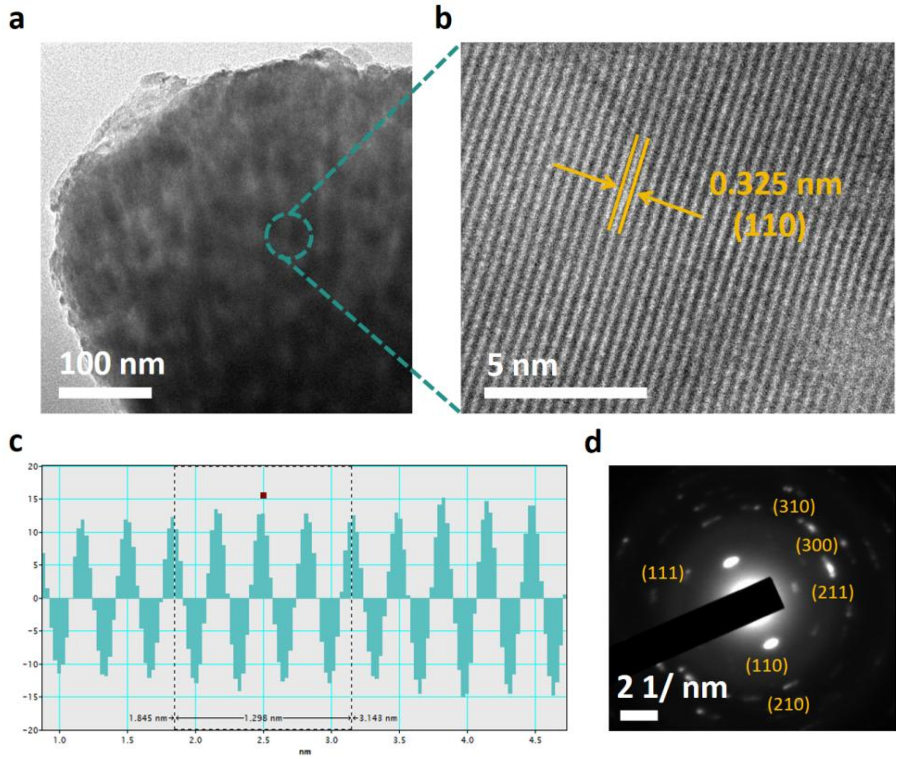

Figure S3. (a) Magnified TEM image, (b) HRTEM image, (c) corresponding IFFT image and (d) SAED pattern of meso-TiO_2 microspheres after calcination at 400 °C.

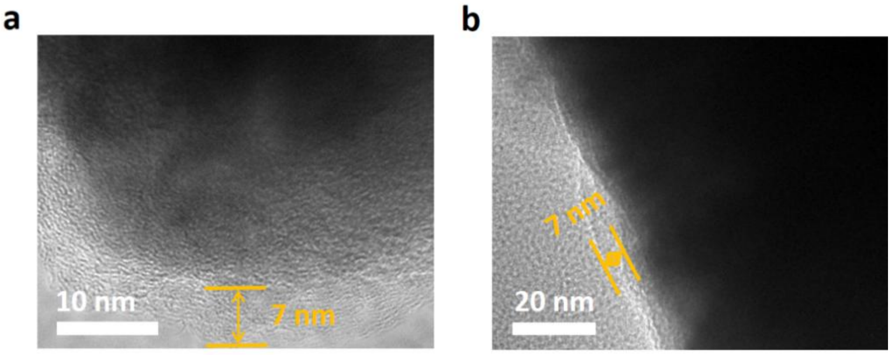

Figure S4. TEM images with different magnifications and structure model of the meso-TiO_2@PPy-7 microspheres.

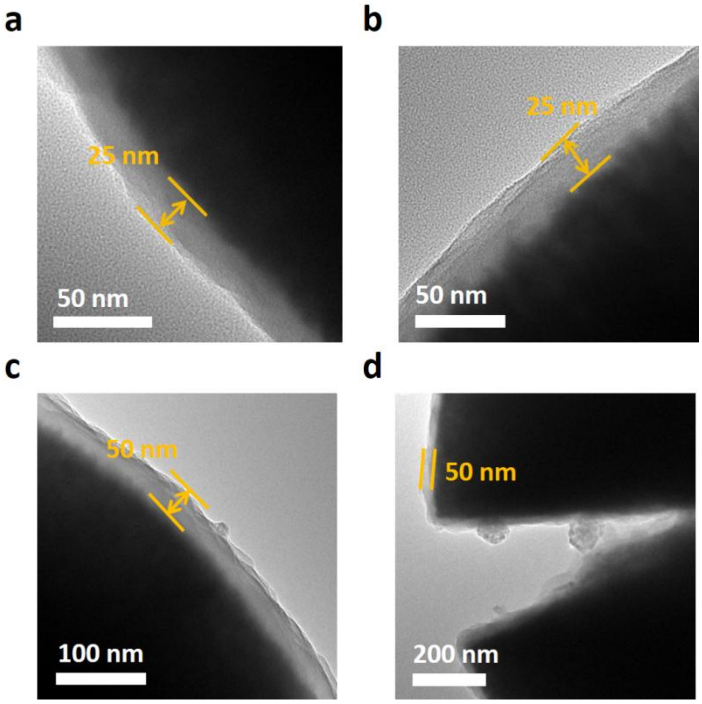

Figure S5. TEM images with different magnifications of (a, b) the meso-TiO_2@PPy-25 and (c, d) the meso-TiO_2@PPy-50 microspheres.

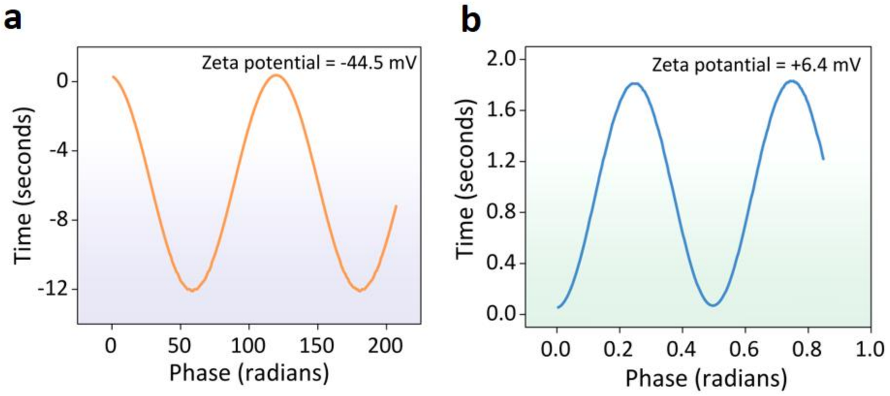

Figure S6. Zeta potentials of (a) meso-TiO_2 and (b) meso-TiO_2@PPy-7 at pH=7.

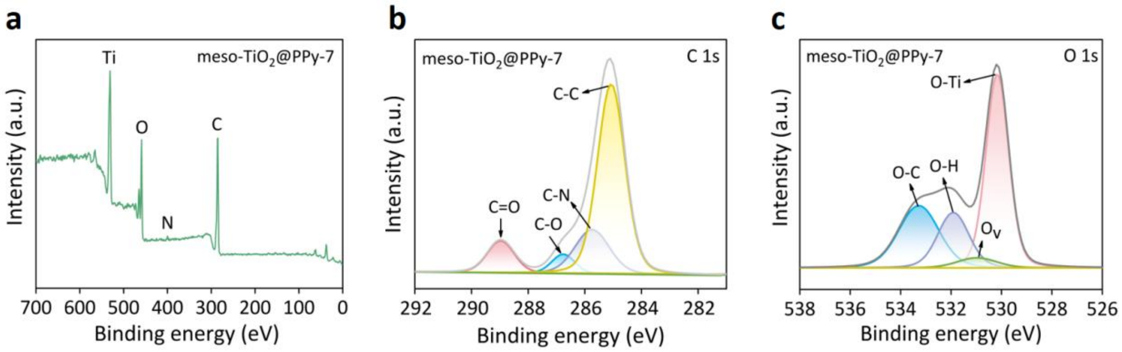

Figure S7. (a) The full-scale XPS spectrum for the meso-TiO_2@PPy-7 sample. (b) High-resolution C 1s XPS spectra for the meso-TiO_2@PPy-7 sample. (c) High-resolution O 1s XPS spectra for the meso-TiO_2@PPy-7 sample.

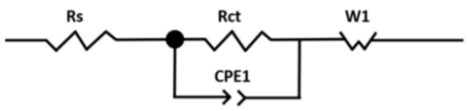

Figure S8. The fitting equivalent-circuit diagram.

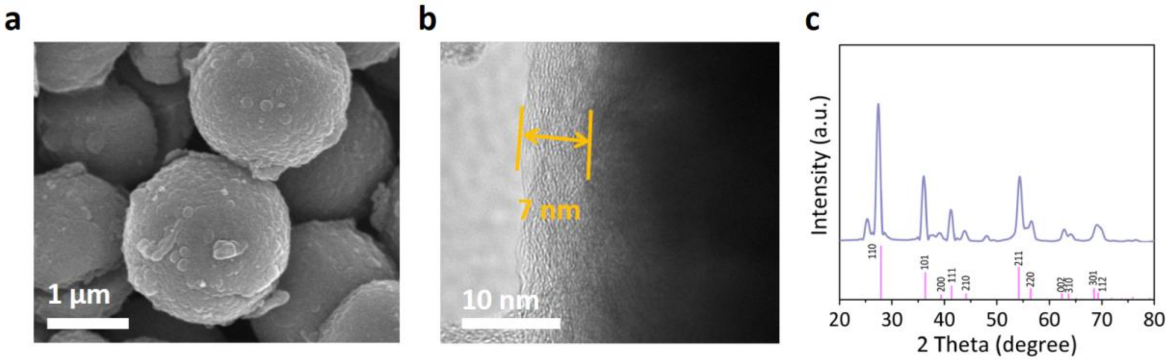

Figure S9. (a) The SEM image and (b) TEM image of the meso-TiO_2@PPy-7 microspheres anode after 2000 cycles at 1 A g^{-1} by washing with glycol solvent. (c) XRD pattern of the meso-TiO_2@PPy-7 sample after 2000 cycles at 1 A g^{-1}.

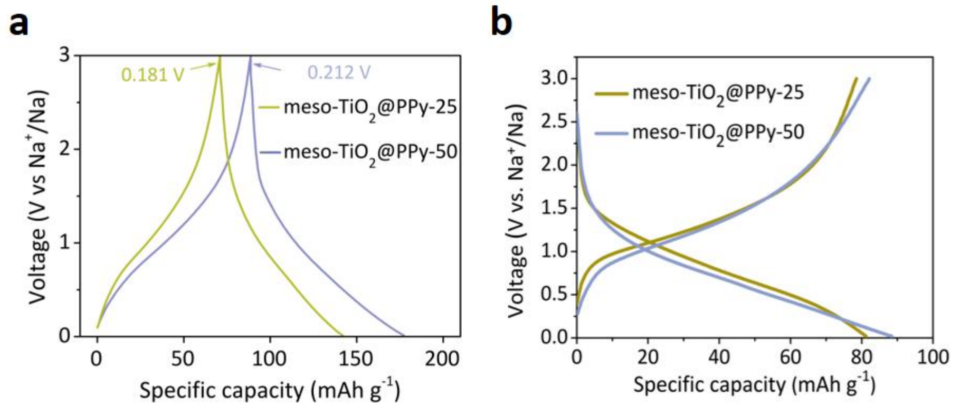

Figure S10. Electrochemical measurements of the meso-TiO_2@PPy-25 and meso-TiO_2@PPy-50 anodes. (a) Galvanostatic charge-discharge profile of the meso-TiO_2@PPy-25 and meso-TiO_2@PPy-50 electrodes at 1 A g^{-1}. (b) Charge-discharge curves of the meso-TiO_2@PPy-25 and meso-TiO_2@PPy-50 electrodes at 1 A g^{-1} at initial cycles.

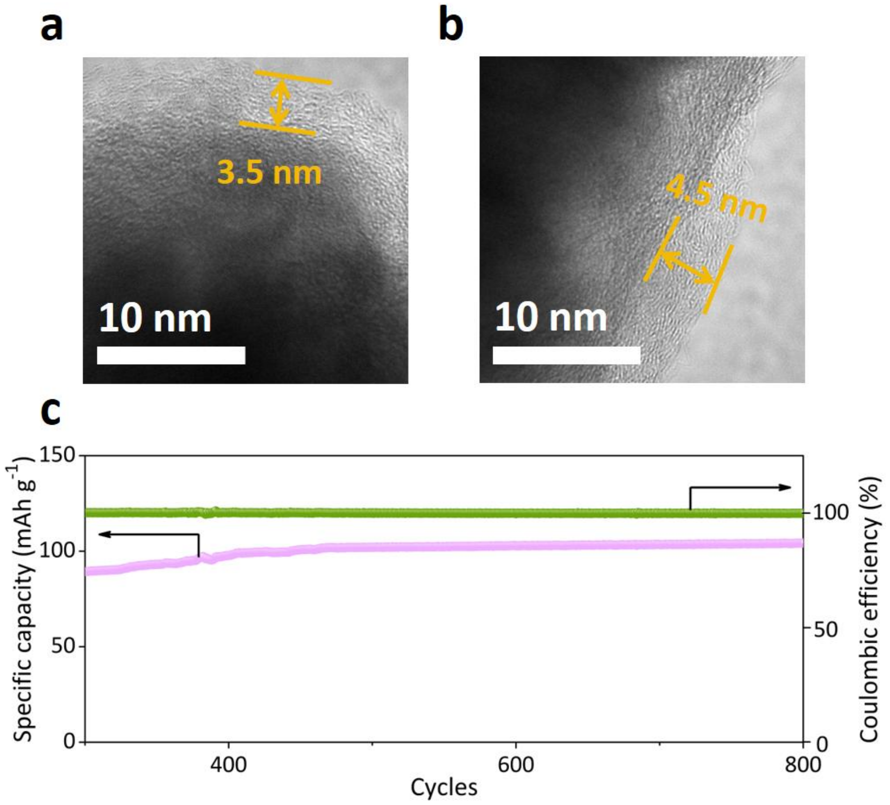

Figure S11. (a, b) TEM images of the meso-TiO_2@PPy-4 sample. (c) Cycling stability of the meso-TiO_2@PPy-4 electrode at 1.0 A g^{-1}.

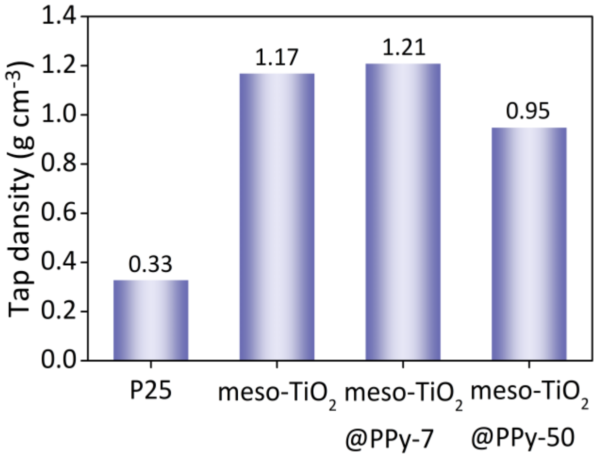

Figure S12. Summarized tap densities of different nanostructured TiO_2 samples, including commercial P25 nanoparticles, meso-TiO_2 microspheres, meso-TiO_2@PPy-7 microspheres, and meso-TiO_2@PPy-50 microspheres.

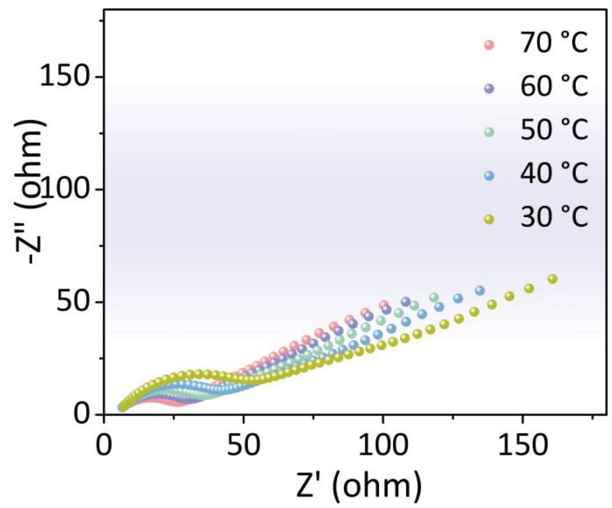

Figure S13. The Nyquist plots of meso-TiO_2@PPy-7 at different temperatures.

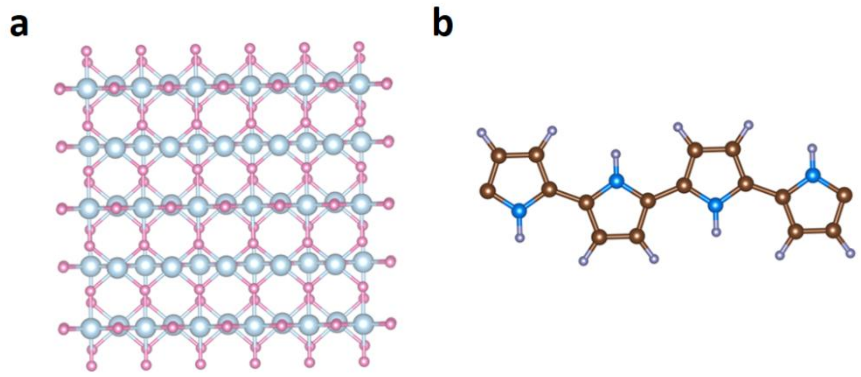

Figure S14. Structure models of (a) meso-TiO_2 and (b) PPy. Color code: Ti (cyan), O (pink), H (purple), N (dark blue), and C (brown).

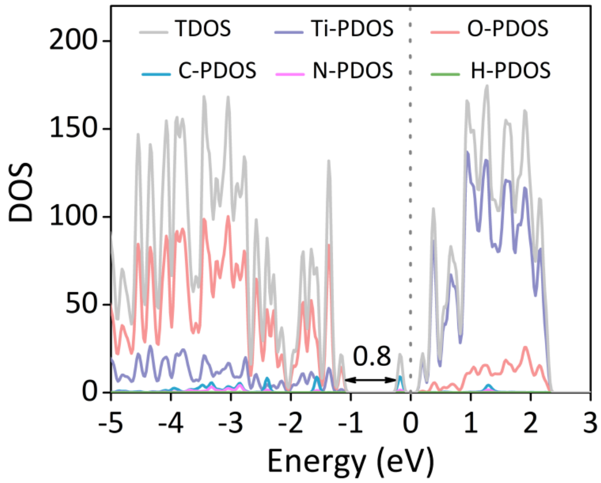

Figure S15. The calculated total density of states (TDOS) and partial density of states (PDOS) of the meso-TiO_2@PPy.

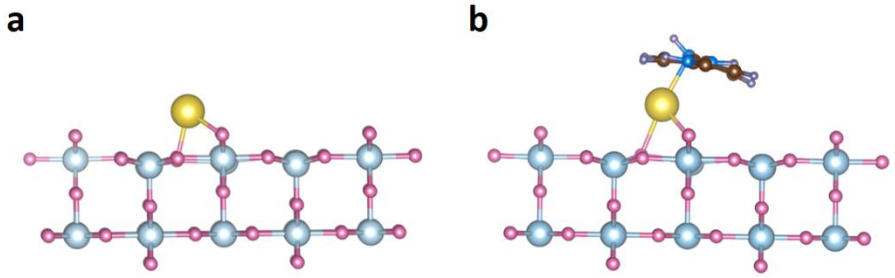

Figure S16. Structure models of Na^+ adsorption on (a) meso-TiO_2 and (b) meso-TiO_2@PPy. Color code: Ti (cyan), O (pink), H (purple), N (dark blue), C (brown), and Na (yellow).

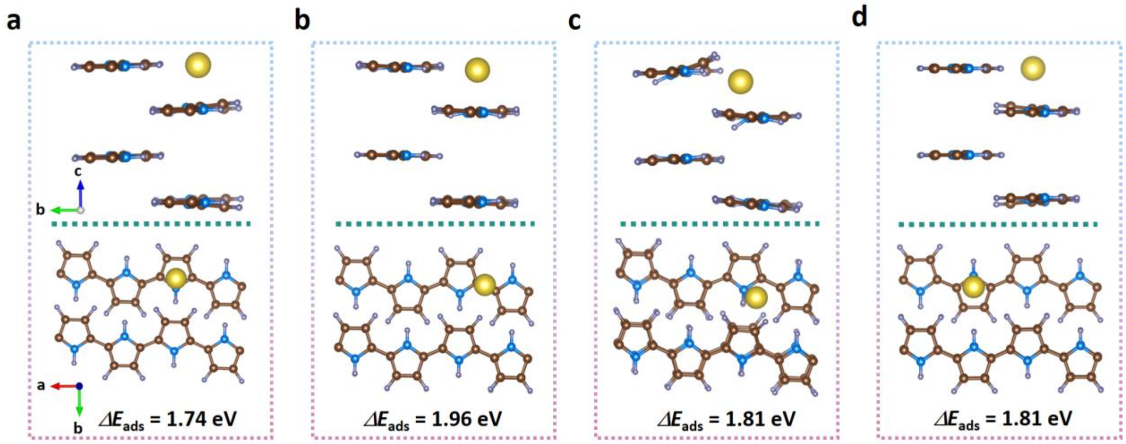

Figure S17. (a-d) Several structural models of Na^+ adsorption on the meso-TiO_2@PPy with 4-layer PPy. Color code: H (purple), N (dark blue), C (brown), and Na (yellow).

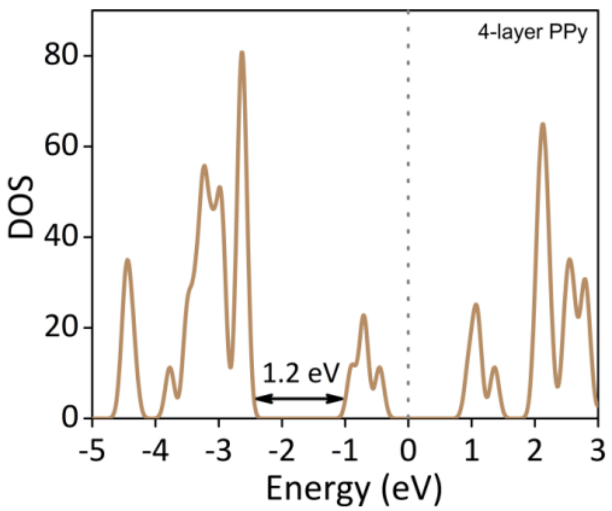

Figure S18. The calculated total density of states (TDOS) of the meso-TiO_2@PPy with 4-layer PPy.

Table S1. Summary of the physicochemical properties of the meso-TiO_2, meso-TiO_2@PPy-7, and commercial P25.

Sample	S_{BET} ($m^2\ g^{-1}$)	Pore size (nm)	V_t ($cm^3\ g^{-1}$)	Phase	PPy weight
meso-TiO_2@PPy-7	144	3.2	0.41	R	4.8%
meso-TiO_2	119	3.5	0.39	R	0
P25[8]	45	17.8	0.24	79%A, 21%R	0

S_{BET}: surface area calculated from the Brunauer-Emmett-Teller (BET) method using nitrogen adsorption isotherms. V_t: total pore volume. The weight ratio of each composition is calculated from the TG curves.

Table S2. A survey of electrochemical properties of representative TiO_2-based anode materials.

Materials	Electrolyte[a]	Potential (V vs. Na^+/Na)	Current density (A g^{-1})	Cycle numbers	Capacity (mAh g^{-1})	Ref.
meso-TiO_2@PPy	1 M $NaPF_6$ in DGDE	0.01~3.0	1.0	2000	160.6	**This work**
meso-TiO_2@meso-C	1 M $NaPF_6$ in DGDE	0.01~3	1.0	5000	115	[8]
meso-TiO_2 microsphere	1 M $NaPF_6$ in DGDE	0.01~3.0	1.0	5000	130	[9]
N-doped mesoporous TiO_2 nanofiber	1 M $NaPF_6$ in EC/DEC	0.001~3.0	1.67	500	148	[10]
olive-like anatase TiO_2@C	1 M $NaClO_4$ in FEC	0.01~2.5	3.36	1000	96	[11]
TiO_2/Graphene	1 M $NaClO_4$ in EC/PC	0.05~3.0	0.5	4300	120	[12]
TiO_2(B) bunchy hierarchical structure	1 M $NaClO_4$ in EC/DMC	0~2.5	0.1675	100	167	[13]
N-doped carbon coated mesoporous TiO_2 nanotube	1 M $NaClO_4$ in EC/DMC	0.01~3.0	0.67	2200	158	[14]
amorphous TiO_2 inverse opal	1 M $NaClO_4$ in PC	0.01~3.0	0.5	500	130	[15]
TiO_2 nanosheets	1 M $NaClO_4$ in EC/DMC	0.01~2.5	0.2	700	200	[16]
TiO_2 microfibers@C	1 M $NaClO_4$ in EC/PC/FEC	0.1~2.0	0.05	500	167	[17]

[a]The abbreviation of EC, PC, DMC, FEC, DEC, and DGDE represent ethylene carbonate, propylene carbonate, dimethyl carbonate, fluoroethylene carbonate, diethyl carbonate, and diethylene glycol dimethyl, respectively.

References

(1) Liu, Y.; Che, R.; Chen, G.; Fan, J.; Sun, Z.; Wu, Z.; Wang, M.; Li, B.; Wei, J.; Wei, Y.; Wang, G.; Guan, G. Z.; Elzatahry, A. A.; Bagabas, A. A.; Al-Enizi, A. M.; Deng, Y. H.; Peng, H. S.; Zhao, D. Y. Radially oriented mesoporous TiO_2 microspheres with single-crystal-like anatase walls for high-efficiency optoelectronic devices. *Sci. Adv.* **2015**, *1*, e1500166.

(2) Long, B.; Qiao, Z.; Zhang, J.; Zhang, S.; Balogun, M. S.; Lu, J.; Song, S.; Tong, Y. Polypyrrole-encapsulated amorphous Bi_2S_3 hollow sphere for long life sodium ion batteries and lithium–sulfur batteries. *J. Mater. Chem. A.* **2019**, *7*, 11370–11378.

(3) Li, W.; Yang, J. P.; Wu, Z. X.; Wang, J. X.; Li, B.; Feng, S. S.; Deng, Y. H.; Zhang, F.; Zhao, D. Y. A versatile kinetics-controlled coating method to construct uniform porous TiO_2 shells for multifunctional core−shell structures. *J. Am. Chem. Soc.* **2012**, *134*, 11864−11867.

(4) Blöchl, P. E. Projector augmented-wave method. *Phys. Rev. B* **1994**, *50*, 17953.

(5) Kresse, G.; Hafner, J. Ab initio molecular dynamics for liquid metals. P*hys. Rev. B* **1993**, *47*, 558.

(6) Perdew, J. P.; Burke, K.; Ernzerhof, M. Generalized gradient approximation made simple. *Phys. Rev. Lett.* **1996**, *77*, 3865.

(7) Kim, H. S.; Cook, J. B.; Lin, H. K.; Jesse, S.; Sarah, H.; Ozolins, V.; Dunn, B. Oxygen vacancies enhance pseudocapacitive charge storage properties of MoO_{3-x}. *Nat. Mater*. **2017**, *16*, 454–460.

(8) He, Y.; Miao, X.; Wang, W.; Li, J.; Zhang, J.; Li, R.; Yang, L.; Liu, L.; Wang, Y.; Guo, Z.; Zhao, D.; Lan, K. High-volumetric pseudocapacitive sodium storage in densely packed mesoporous titanium dioxide-carbon composite. *Cell. Rep. Phys. Sci*. **2024**, *5*, 102123.

(9) Lan, K.; Liu, L.; Zhang, J. Y.; Wang, R.; Zu, L.; Lv, Z.; Wei, Q.; Zhao, D. Precisely designed mesoscopic titania for high-volumetric-density pseudocapacitance. *J. Am. Chem. Soc.* **2021**, *143*, 14097–14105.

(10) Wu, Y.; Liu, X. W.; Yang, Z. Z.; Gu, L.; Yu, Yan. Nitrogen-doped ordered mesoporous anatase TiO_2 nanofibers as anode materials for high performance sodium-ion batteries. *Small* **2016**, *12*, 3522–3529.

(11) Chen, J.; Zhang, Y.; Zou, G. Q.; Huang, Z. D.; Li, S. M.; Liao, H. X.; Wang, J. F.; Hou, H. S.; Ji, X. B. Size-tunable olive-like anatase TiO_2 coated with carbon as superior anode for sodium-ion batteries. *Small* **2016**, *12*, 5554–5563.

(12) Chen, C. J.; Wen, Y. W.; Hu, X. L.; Ji, X.L.; Yan, M. Y.; Mai, L. Q.; Hu, P.; Shan, B.; Huang, Y. H. Na^+ intercalation pseudocapacitance in graphene-coupled titanium oxide enabling ultra-fast sodium storage and long-term cycling. *Nat. Commun.* **2015**, *6*, 6929.

(13) Liu, S.; Niu, K.; Chen, S. L.; Sun, X.; Liu, L. H.; Jiang, B.; Chu, L. H.; Lv, X. J.; Li, M. C. TiO_2 bunchyhierarchical structure with effective enhancement in sodium storage behaviors. *Carbon Energy*. **2022**, *4*, 645–653.

(14) Li, B. S.; Anwer, S.; Huang, X. H.; Luo, S. H.; Fu, J.; Liao, K. Nitrogen-doped carbon encapsulated in mesoporous TiO_2 nanotubes for fast capacitive sodium storage. *J. Energy. Chem.* **2021**, *55*, 202–210.

(15) Zhou, M.; Xu, Y.; Wang, C. L.; Li, Q. W.; Xiang, J. X.; Liang, L. Y.; Wu, M. H.; Zhao, H. P.; Lei, Y. Amorphous TiO_2 inverse opal anode for high-rate sodium ion batteries. *Nano Energy* **2017**, *31*, 514–524.

(16) Shoaib, A.; Huang, Y. X.; Liu, J.; Liu, J. J.; Xu, M.; Wang, Z. H.; Chen, R. J.; Zhang, J. T.; Wu, F. Ultrathin single-crystalline TiO_2 nanosheets anchored on graphene to be hybrid network for high-rate and long cycle-life sodium battery electrode application. *J. Power Sources* **2017**, *342*, 405–413.

(17) Wang, N.; Gao, Y.; Wang, Y. X.; Liu, K.; Lai, W. H.; Hu, Y. M.; Zhao, Y.; Chou, S. L.; Jiang, L. Nanoengineering to achieve high sodium storage: A case study of carbon coated hierarchical nanoporous TiO_2 microfibers. *Adv. Sci.* **2016**, *3*, 1600013.
